# Rateless Coded Uplink Transmission Design for Multi-User C-RAN

**DOI:** 10.3390/s19132978

**Published:** 2019-07-05

**Authors:** Yu Zhang, Jiali Xu, Hong Peng, Weidang Lu, Zhaoyang Zhang

**Affiliations:** 1 College of Information Engineering, Zhejiang University of Technology, Hangzhou 310023, China; 2 Zhejiang Provincial Key Laboratory of Information Processing, Communication and Networking, Hangzhou 310027, China; 3 College of Information Science and Electronic Engineering, Zhejiang University, Hangzhou 310027, China

**Keywords:** C-RAN, uplink transmission, rateless code, block fading, degree profile optimization

## Abstract

Cloud radio access network (C-RAN) is a promising technology for the Internet of Things (IoT). In C-RAN, the remote radio head (RRH) and baseband unit (BBU) in the conventional base station are separated, and each BBU is backward centralized into a virtual BBU pool. In this paper, we consider the uplink transmission for the two-user C-RAN with two RRHs under a block fading channel. A novel rateless coded transmission scheme is designed. During each transmission round, each user keeps transmitting to the RRHs using Raptor code until the BBU pool feeds back an acknowledgement (ACK). With the proposed scheme, each user does not require the instant channel state information, which greatly reduces the system overhead. We also design the quantizer at the RRHs and the iterative multi-user detector and decoder at the BBU pool, based on the belief propagation (BP) algorithm. For the Raptor code applied at each user, we optimize the corresponding output node degree profile, based on extrinsic information transfer (EXIT) analysis for the decoding process at the BBU pool. The resulted degree profiles are optimal in an average sense under all possible channel states. The simulation results show that the rateless coded transmission scheme with the optimized degree profiles outperforms the benchmark degree profile in both bit error rate and average system throughput. Moreover, the achieved performance is close to the theoretical limit.

## 1. Introduction

The Internet of Things (IoT) is the trend of the next generation of communication networks. To achieve the IoT, information collection needs to be completed by massive sensors [1]. The International Data Corporation predicts that by 2020, the number of internet-connected devices in the IoT will grow to 50 billion [2]. The large amount of data generated by sensors and devices will create huge network traffic, which brings great challenges to the traditional wireless access networks. On the other hand, it has been noted in [3,4,5,6] that cloud radio access network (C-RAN) is a promising wireless network architecture which can adapt to the rapidly growing IoT traffic and improve the Quality of Service (QoS).

C-RAN is characterized by the separation of the remote radio head (RRH) and the baseband unit (BBU) of each access point in the network. In C-RAN, each RRH is located closer to the user, while each BBU is backward centralized into a virtual BBU pool. The BBU pool and RRHs are connected by high-speed fronthaul links. Since joint signal processing for multiple RRHs can be realized at the BBU pool, C-RAN can achieve a considerably higher spectrum efficiency compared with the traditional cellular networks, and C-RAN can also improve network resource utilization, reduce interference, and reduce energy consumption and overall hardware costs [7,8].

### 1.1. Motivation and Related Works

There exist several issues for the implementation of C-RAN. Firstly, the limited fronthaul link capacity imposes restriction on the data rate between RRHs and the BBU pool. Therefore, signal compressors are necessary at both sides [9,10,11,12]. Secondly, the additional latency introduced by fronthaul will cause stricter restriction on the decoding time at the BBU pool under the conventional hybrid automatic repeat-request (HARQ) framework [13]. Thirdly, for joint signal processing at the BBU pool, global information about the network states and channel states is needed. In consideration of the system overhead, it is nearly infeasible for the transmitter to obtain instantaneous global channel state information. This poses challenges for the application of traditional HARQ-based fixed-rate channel coding (such as low density parity check (LDPC) code and Turbo code) in C-RAN, with which the coding rate of the transmitter is chosen based on the current channel state feedback by the receiver, and the decoding time at the receiver is restricted in order to support HARQ [13]. On the other hand, rateless code (such as Luby transform (LT) code and Raptor code [14]) can encode the information into a codeword of infinite length. With rateless code, the transmitter continuously sends the codeword to the receiver until the latter feeds back an ACK. The performance of rateless code depends on the so-called output node degree profile [14]. The optimized rateless code can perform close to the channel capacity even without instant channel state information [15]. The above properties make rateless code a promising channel coding candidate for C-RAN.

C-RAN has been widely investigated, and there has been a plenary of work on various aspects of C-RAN, e.g., [16,17,18,19,20,21,22,23,24,25,26,27,28,29,30,31]. The network structure and the function split between RRHs and the BBU pool in C-RAN were discussed in [16,17,18]. Literature works [10,20] investigated resource allocation and management in C-RAN. Non-orthogonal multiple access (NOMA) was considered in C-RAN [21,22], where energy efficiency was analyzed and optimized. As for the physical layer, existing work mainly focused on the compressor and pre-coder design at the RRHs, wherein the signal dependence at the RRHs can be utilized to increase the compression rate [23,24,25], and the compressor and pre-coder can be jointly optimized for a higher system achievable rate [26,27,28]. Considering the channel coding in C-RAN, authors in [29,30,31] made efforts on the design and implementation of fixed-rate code, i.e., Turbo code and LDPC code, and the performances of decoding algorithms were evaluated on cloud computing platforms. On the other hand, rateless code, such as LT code and Raptor code, has been studied in various communications systems, including wireless broadcast systems [32], distributed antenna systems [33,34] and relay systems [35,36], but these cannot be directly applied to C-RAN. The application of rateless code as channel code in C-RAN has not been discussed until recently [37,38,39]. In [37], we considered Raptor code for the multi-user downlink of C-RAN, where both the precoder and the degree profile of Raptor code at the BBU pool are jointly optimized under an additive white Gaussian noise (AWGN) channel. In [38,39], a rateless coded transmission scheme was designed for single-user uplink C-RAN, and the degree profile of the user was optimized under both AWGN and a block fading channel.

### 1.2. Contributions

In this paper, we consider the design of a rateless coded transmission scheme for two-user uplink in C-RAN under a block fading channel. It should be noted that in the proposed scheme, instant channel state information (CSI) for each user is not required. For each transmission round, each user encodes its own message using Raptor code with a pre-optimized degree profile which is fixed over all transmission rounds, and simultaneously transmits to the RRHs. Each RRH compresses its received signals and uploads to the BBU pool for joint signal processing. Upon the BBU pool recovering the messages of both users and feeding back an ACK, the current transmission round ends. We summarize the main contributions of this paper as follows:

(1) We propose a rateless coded uplink transmission scheme for two-user C-RAN with two RRHs, including the quantizer at each RRH and the iterative multi-user detector and decoder based on the belief propagation (BP) algorithm at the BBU pool.
(2) We resort to extrinsic information transfer (EXIT) to analyze the iterative detecting and decoding process at the BBU pool. Based on this, the condition for successfully decoding is derived.
(3) Based on the EXIT analysis, we optimize the degree profiles of the Raptor code for each user. Explicitly, we search the optimal degree profiles to minimize the threshold signal-to-noise ratio (SNR) under a fixed average Raptor code length and the condition of successfully decoding over all possible channel states. Therefore, the resulted degree profiles are optimal in an average sense over all possible channel states.

Note that compared with the single-user case in [38,39], rateless coded transmission design for multi-user C-RAN uplink is more challenging. Firstly, the receiver structure for a multi-user case is more complicated than for a single-user case, wherein joint de-quantization, multi-user detection and channel decoding should be performed at the BBU pool. Secondly, the rateless code profile optimization scheme for the single-user case cannot be directly adopted here, due to the fact that the EXIT of the decoding process is totally different from the single-user case.

Organization: This paper is organized as follows. In Section 2, we introduce the system model. The rateless coded uplink transmission scheme is presented in Section 3. In Section 4, EXIT analysis and degree profile optimization are discussed. Simulation results are shown in Section 5, and Section 6 concludes the paper.

## 2. System Model

**Figure 1:**
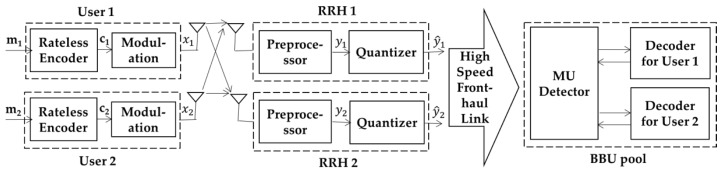
Rateless coded transmission for two-user uplink in cloud radio access network (C-RAN) with two remote radio heads (RRHs).

The considered uplink C-RAN system is depicted in Figure 1, which consists of two users, two RRHs, and the BBU pool. Both users and both RRHs are equipped with a single antenna. For the wireless link from user i, i=1,2, to RRH j, j=1,2, the channel gain is denoted as $h_{ji}$.

We assume that the wireless links experience block fading. The channel gain of each link remains constant during one round of transmission, but independently and randomly changes round by round, satisfying some probability distribution (e.g., Rayleigh distribution). As for the receiver side, the BBU pool has the knowledge of the instant channel gain between each user and RRH. On the other hand, the user is not required to be aware of the instant channel information. During each transmission round, the two users continuously transmit to the BBU pool until the latter recovers the messages and feeds back an ACK. The detailed procedure of each transmission round is as follows.

Firstly, user i, i=1,2, encodes its message $m_{i}$ of length K using Raptor code to obtain the coded bits $c_{i}$ and then modulates the bits into symbols $x_{i}$, which are sent to the RRHs. Note that the degree profiles used for Raptor encoding are fixed over all transmission rounds. At each RRH, the received signals are preprocessed into the baseband signals, which can be expressed as

(1) $$y=H\sqrt{P}x+n,$$

where $y≜{\left[y_{1},y_{2}\right]}^{T},\;H≜{\left[h_{ji}\right]}_{2\times 2}$, $x≜{\left[x_{1},x_{2}\right]}^{T}$, $n≜{\left[n_{1},n_{2}\right]}^{T}$, $y_{j},\;j=1,2,$ is the base band signal at RRH *j*, P is the transmission power of each user, $n_{j}$ is the independent additive white Gaussian noise at RRH *j* with a mean of zero and variance of $\sigma_{0}^{2}$.

Then, RRH *j* quantizes the baseband signals to meet the fronthaul link capacity restriction. The quantized signals ${\hat{y}}_{j}$ are uploaded to the BBU pool through the fronthaul link. At the BBU pool, an iterative receiver structure with a multi-user detector (MU detector) and a channel decoder based on the belief propagation (BP) algorithm is applied. When the messages from both users are successfully recovered, the BBU pool feeds back the ACK via RRHs to inform the users to stop transmitting.

## 3. Rateless Coded Uplink Transmission Scheme

In this section, we present the detailed uplink transmission scheme based on rateless code. Firstly, we show the encoding process at the users. Then, the quantization scheme at the RRHs is given. Finally, we propose the iterative detecting and decoding algorithm at the BBU pool.

### 3.1. Rateless Encoder at the User

Each user applies Raptor code to encode its intended messages. Explicitly, to generate the coded bits $c_{i}$ at each user *i*, the message $m_{i}$ is firstly encoded by LDPC code with rate $R_{p}$, and then by LT code with an output node degree profile:

(2) $$\Omega_{i}\left(x\right)=\sum_{d=1}^{d_{c}}\Omega_{i,d}x^{d},\;i=1,2,$$

where $d_{c}$ is the maximum output node degree and $\Omega_{i,d}$ is the probability that a coded bit $c_{i}$ (also named ‘output bit’ in the following) is with degree d.

Each output bit is generated as follows. The degree of each output bit is randomly chosen according to the degree profile (2). For an output bit with degree d, d bits are randomly picked from the LDPC coded message (also named ‘input bits’ in the following). These d bits are XORed and the result is the value of this output bit. Through the above encoding process, the output bits can be generated infinitely. As for the modulation, for simplicity, we apply binary modulation, i.e., bits 0 and 1 are mapped to 1 and −1, respectively. The users continuously send the modulated signals until an ACK is received.

### 3.2. Scalar Quantizer at the RRH

In order to meet the limited fronthaul capacity, the baseband signal should be compressed at the RRH before being uploaded to the BBU pool. Here, we use a scalar quantizer with uniform quantization intervals. The number of quantization bits is determined by the fronthaul capacity limitation. Due to the concern of practicality, the quantization thresholds only depend on the number of quantization bits and the statistics of the channel gain.

According to (1), the base band signal at RRH *j* is

(3) $$y_{j}=h_{j1}\sqrt{P}x_{1}+h_{j2}\sqrt{P}x_{2}+n_{j},\;j=1,2,$$

whose variance is calculated by:

(4) $$D\left(y_{j}\right)=E\left(y_{j}^{2}\right)-E^{2}\left(y_{j}\right)=P\sigma_{j1}^{2}+P\sigma_{j2}^{2}+\sigma_{0}^{2},$$

where $\sigma_{ji}^{2}$ is the variance of the channel gain from user *i* to RRH *j*.

According to [40], it can be regarded that the value of $y_{j}$ is almost distributed in the range of $\left(-3\sqrt{D\left(y_{j}\right)},3\sqrt{D\left(y_{j}\right)}\right)$. Let the number of quantization bits be b and $M=2^{b}$. Then, the signal $y_{j}$ at RRH *j* is quantized into ${\hat{y}}_{j}$ according to the following rule:

(5) $${\hat{y}}_{j}=Q_{j}\left(y_{j}\right)=\{\begin{array}{l} q_{j1},\;-\infty <y_{j}<-3\sqrt{D\left(y_{j}\right)}+\Delta_{j}\\ q_{jk},-3\sqrt{D\left(y_{j}\right)}+\left(k-1\right)\Delta_{j}\leq y_{j}<-3\sqrt{D\left(y_{j}\right)}+k\Delta_{j},\;k=2,\ldots ,M-1\\ q_{jM},3\sqrt{D\left(y_{j}\right)}-\Delta_{j}\leq y_{j}<\infty \end{array},$$

where $\Delta_{j}=\frac{3\sqrt{D\left(y_{j}\right)}}{M}$ is the quantization interval and $q_{jk}=-3\sqrt{D\left(y_{j}\right)}+\left(\;k-\frac{1}{2}\right)\Delta ,\;k=1,\ldots ,M$, for the quantizer at RRH *j*.

### 3.3. Iterative Detecting and Decoding at the BBU Pool

**Figure 2:**
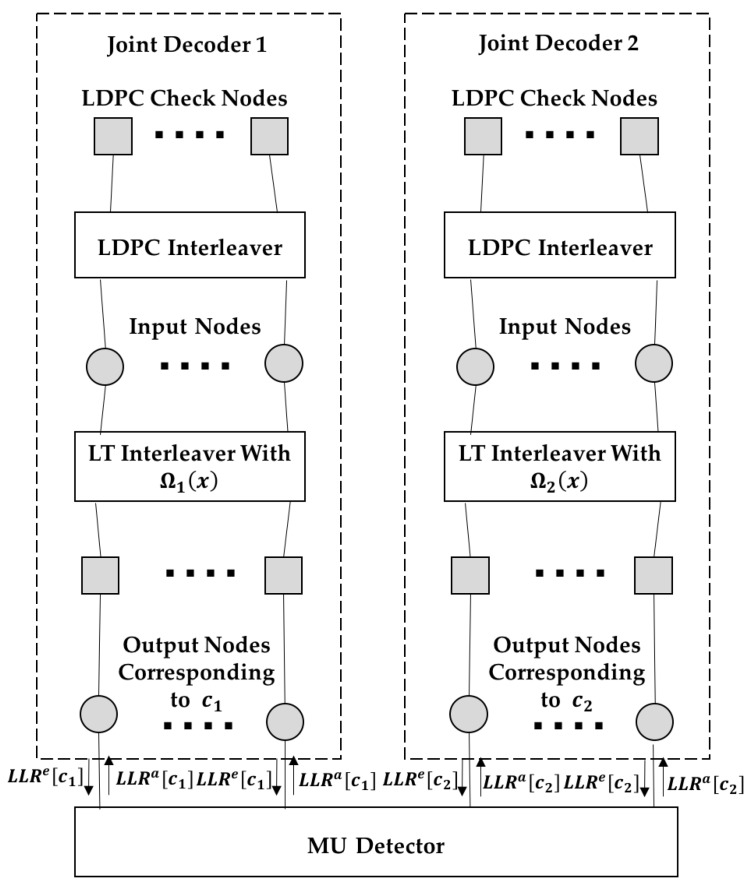
Decoding graph at the baseband unit (BBU) pool.

Figure 2 shows the decoding graph corresponding to the iterative detector and decoder at the BBU pool. Square nodes represent check nodes, and circular nodes represent variable nodes which are also named as input nodes or output nodes, as shown in the figure. The left and right subgraphs in the upper part of the figure represent the Raptor decoding graph for the messages from user 1 and user 2, respectively. The input nodes and output nodes in the Raptor decoding graph represent the corresponding input bits (LDPC coded bits) and output bits (Raptor coded bits), respectively. For simplicity, each output node and the associated LT check node are regarded as a whole and referred to as “output node” in the following. The lower part of the figure is the multi-user detector. $LLR^{a}\left[c_{i}\right]$ and $LLR^{e}\left[c_{i}\right]$ denote the log-likelihood-ratio (LLR) of the output bits of user *i* exchanged between the MU detector and decoders.

The MU detector calculates the LLRs of the output bits $c_{i}$ of user *i*, *i* = 1, 2, according to the quantized signals ${\hat{y}}_{1}$ and ${\hat{y}}_{2}$ from both RRHs and the soft outputs $LLR^{e}\left[c_{i}\right]$ from the decoders. The LLR of each output bit for user *i* is calculated by:

(6) $$\begin{array}{l} LLR^{a}\left[c_{i}\right]=\ln\frac{P\left(c_{i}=0|{\hat{y}}_{1},{\hat{y}}_{2}\right)}{P\left(c_{i}=1|{\hat{y}}_{1},{\hat{y}}_{2}\right)}\\ =\ln\frac{P\left({\hat{y}}_{1},{\hat{y}}_{2}|c_{i}=0,c_{i^{\prime }}=1\right)+\frac{P\left(c_{i^{\prime }}=0\right)}{P(c_{i^{\prime }}=1)}P\left({\hat{y}}_{1},{\hat{y}}_{2}|c_{i}=0,c_{i^{\prime }}=0\right)}{P\left({\hat{y}}_{1},{\hat{y}}_{2}|c_{i}=1,c_{i^{\prime }}=1\right)+\frac{P\left(c_{i^{\prime }}=0\right)}{P(c_{i^{\prime }}=1)}P\left({\hat{y}}_{1},{\hat{y}}_{2}|c_{i}=1,c_{i^{\prime }}=0\right)}\cdot \frac{P\left(c_{i}=0\right)}{P\left(c_{i}=1\right)}\;i\\ =1,2,i^{\prime }=3-i, \end{array}$$

where $\frac{P\left(c_{i}=0\right)}{P\left(c_{i}=1\right)}$ equals 1, and the value of $\frac{P\left(c_{i^{\prime }}=0\right)}{P(c_{i^{\prime }}=1)}$ is 1 at the beginning of decoding and equals $e^{LLR^{e}\left[c_{i^{\prime }}\right]}$ during the decoding iteration, in which $LLR^{e}\left[c_{i^{\prime }}\right]$ is the soft output from the Raptor decoder for the other user *i*’ in the previous decoding iteration. Taking the case of $i=1,\;c_{i}=0,\;c_{i^{\prime }}=1$ as an example, the conditional probability $P\left({\hat{y}}_{1},{\hat{y}}_{2}|c_{1}=0,c_{2}=1\right)$ in (6) is calculated as follows. Note that when fixing $\;c_{i}=0,\;c_{i^{\prime }}=1$, the received signals $y_{1}$ and $y_{2}$ at the RRHs are Gaussian distributed. According to (5), we can derive:

(7) $$P\left({\hat{y}}_{1},{\hat{y}}_{2}|c_{1}=0,c_{2}=1\right)=\int_{k_{11}}^{k_{12}}\frac{1}{\sqrt{2\pi \sigma_{0}^{2}}}e^{-\frac{{\left(x-h_{11}+h_{12}\right)}^{2}}{2\sigma_{0}^{2}}}dx\cdot \int_{k_{21}}^{k_{22}}\frac{1}{\sqrt{2\pi \sigma_{0}^{2}}}e^{-\frac{{\left(x-h_{21}+h_{22}\right)}^{2}}{2\sigma_{0}^{2}}}dx,$$

where the integral range [$k_{11},k_{12}$] and [$k_{21},k_{22}$] denote the quantization intervals that ${\hat{y}}_{1}$ and ${\hat{y}}_{2}$ represent according to (5), respectively. As an example, if ${\hat{y}}_{1}=q_{11}$, the range for the first integral is $\left(-\infty ,\;-3\sqrt{D\left(y_{1}\right)}+\Delta_{1}\right)$. The other three conditional probability terms can be calculated similarly.

The iterative detecting and decoding is based on the BP algorithm [41], where ‘messages’ are interchanged along the edges between variable nodes and check nodes. Each ‘message’ along the edge is the LLR of the corresponding variable node (representing either the input bits or the output bits), which is connected by this edge. The whole decoding process can be divided into two stages. In the first stage, the decoding iteration is performed on the entire decoding graph (including the MU detector), until the mean LLR of the input nodes of both users exceed a predetermined threshold $m_{th}$, which is the minimum requirement for successfully decoding the rate $R_{p}$ LDPC precoder and can be derived using the method in [31]. In the second stage, decoding iteration is performed individually on the LDPC decoding graph of each user to remove the residual errors. The detailed procedure of the first decoding stage is given in the following.

For the iteration l, firstly, message passing is performed on the Raptor decoding graph of user 1, which is shown in step 1 to step 6.

*Step 1*: The message transferred from an input node *i* to an LDPC check node *c* is given by:

(8) $$m_{ic}^{\left(l\right)}=\sum_{o}m_{oi}^{\left(l-1\right)},$$

where $m_{oi}^{\left(l-1\right)}$ is the message from the LT output node of user 1 to the input node in the previous round l−1.

*Step 2*: The message from an LDPC check node *c* to an input node *i* is given by:

(9) $$\tanh\left(\frac{m_{ci}^{\left(l\right)}}{2}\right)=\prod_{i^{\prime }\neq i}\tanh\left(\frac{m_{i^{\prime }c}^{\left(l\right)}}{2}\right),$$

where $m_{i^{\prime }c}^{\left(l\right)}$ is the message from the input nodes which are connected to the LDPC check node *c* (except the input node i itself).

*Step 3*: The message from an input node *i* to an LT output node *o* is:

(10) $$m_{io}^{\left(l\right)}=\sum_{o^{\prime }\neq o}m_{o^{\prime }i}^{\left(l-1\right)}+\sum_{c}m_{ci}^{\left(l\right)},$$

where the first summation is over all LT output nodes adjacent to i other than o itself, and the second summation is over all LDPC check nodes connected to *i*.

*Step 4*: The message from an LT output node *o* to an input node *i* is given by:

(11) $$\tanh\left(\frac{m_{oi}^{\left(l\right)}}{2}\right)=\tanh\left(\frac{z}{2}\right)\prod_{i^{\prime }\neq i}\tanh\left(\frac{m_{i^{\prime }o}^{\left(l\right)}}{2}\right),$$

where $z=LLR^{a}\left[c_{1}\right]$ is the message from the MU detector and is calculated by (6), and the product is over all input symbols adjacent to o other than i itself.

*Step 5:*$LLR^{e}\left[c_{1}\right]$ transferred to the MU detector is given by:

(12) $$LLR^{e}\left[c_{1}\right]=\sum_{o}m_{oi}^{\left(l-1\right)}+\sum_{c}m_{ci}^{\left(l\right)}.$$

*Step 6:* Update the message at the input code:

(13) $$m_{i}^{\left(l\right)}=\sum_{o}m_{oi}^{\left(l\right)}.$$

Then, a similar decoding procedure is performed on the Raptor decoding graph of user 2, and after that, one decoder iteration is completed.

**Figure 3:**
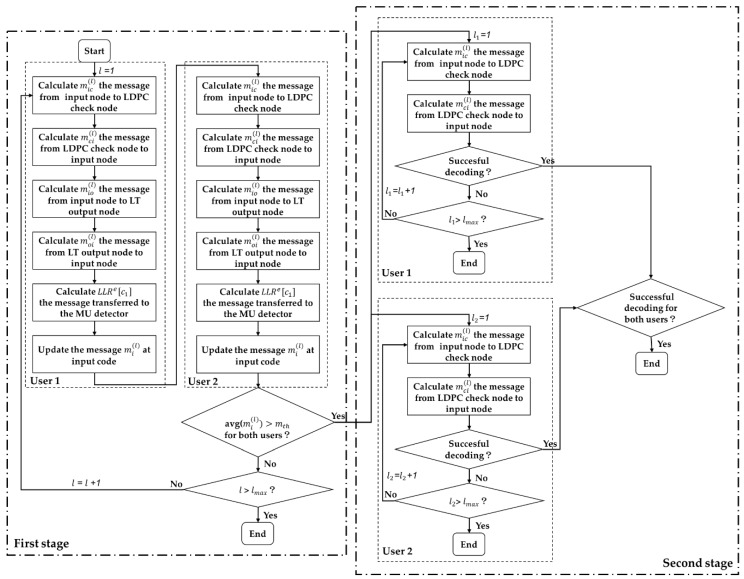
Flow chart of the decoding procedure.

When the mean LLR of the input nodes of both users (as given in (13)) exceeds $m_{th}$, the second stage starts and message exchange will be individually performed on the LDPC decoding graph of each user. The specific process of this stage is similar to Step 1 to Step 2, and is omitted here. Figure 3 is the flow chart of the decoding procedure.

## 4. Decoding Performance Analysis and Degree Profile Design

In this section, we optimize the output degree profile of the Raptor code used by each user to further improve the achieved sum throughput of the system. Note that we assume that each user is not aware of the instantaneous CSI. Hence, it can only be assigned one optimized output degree profile, which is used to generate Raptor code during all rounds of transmission. Therefore, the objective is to find a universally good output degree profile in an average sense for each user under all possible channel states, with the knowledge of CSI statistics, i.e., the probability distribution of the channel state for each link.

### 4.1. EXIT Analysis of the Decoding Process

Before the degree profile optimization, we first analyze the decoding process at the BBU pool to derive the successful decoding condition. The analysis is based on EXIT [42]. Explicitly, we trace the update of the extrinsic information (EI) transferred between output nodes and input nodes in the decoding graph during the decoding iterations.

EI is defined as the mutual information between the LLR messages and the corresponding bits (i.e., input bits and output bits in this paper). It can be assumed that the LLR messages interchanged between the variable nodes and check nodes are symmetrically Gaussian distributed [42]. EI carried by LLR messages with a mean of τ and a variance of 2τ is given by [42]:

(14) $$J\left(\tau \right)=1-\frac{1}{\sqrt{4\pi \tau }}\int_{-\infty }^{\infty }\log_{2}\left(1+e^{-\nu }\right)\exp\left(-\frac{{\left(\nu -\tau \right)}^{2}}{4\tau }\right)d\nu .$$

Before the EXIT analysis, some definitions are given first. Recall the decoding graph in Figure 2. The LDPC variable node degree distribution is defined as $\xi \left(x\right)=\sum_{d=1}^{d_{v}^{\prime }}\xi_{d}x^{d}$, where $\xi_{d}$ is the probability of an LDPC variable node with degree d, and $d_{v}^{\prime }$ is the maximum degree of the variable nodes. (Note that both users employ the same LDPC code). The edge distribution of the LDPC check nodes can be defined as $\tilde{\rho }\left(x\right)=\sum_{d=1}^{d_{c}^{\prime }}\;{\tilde{\rho }}_{d}x^{d-1}$, where ${\tilde{\rho }}_{d}$ is the probability of a randomly chosen edge being connected to a check node with degree d, and $d_{c}^{\prime }$ is the maximum degree of the check nodes. The distribution of the input node degree of user i, i=1,2, is defined as $\alpha_{i}\left(x\right)=\sum_{d=1}^{d_{v}}\alpha_{i,\;d}x^{d},$ $\alpha_{i,d}$ is the probability of the input nodes of user i with degree d, and $d_{v}$ represents the maximum degree of the input nodes. The edge distribution of the input nodes of the user *i* is defined as ${\tilde{\alpha }}_{i}\left(x\right)=\sum_{d=1}^{d_{v}}{\tilde{\alpha }}_{i,d}x^{d-1}$, where ${\tilde{\alpha }}_{i,d}$ is the probability of an edge connected to an input node with degree *d*. Both $\alpha_{i}\left(x\right)$ and ${\tilde{\alpha }}_{i}\left(x\right)$ can be approximated by the Poisson distribution with an average of ${\bar{\alpha }}_{i}$ [43]. The output node edge distribution of user *i* is defined as $\omega_{i}\left(x\right)=\sum_{d=1}^{d_{c}}\omega_{i,d}x^{d-1},$ where $\omega_{i,d}$ represents the probability of an edge connected to the output node with degree d. The relationship between $\omega_{i}\left(x\right)$ and the output node degree distribution $\Omega_{i}\left(x\right)$ given in (2) is given below:

(15) $$\omega_{i}\left(x\right)=\frac{\Omega_{i}^{\prime }\left(x\right)}{\Omega_{i}^{\prime }\left(1\right)}.$$

For ease of reading, we summarize the aforementioned definitions in Table 1, wherein i=1,2.

**Figure 4:**
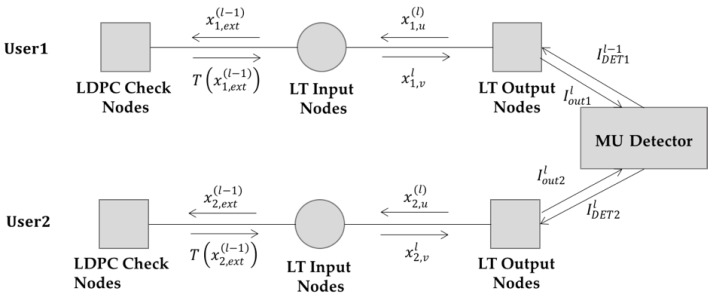
Extrinsic information (EI) transfer on the decoding graph during the decoding iteration.

As shown in Figure 4, we redraw the decoding graph of Figure 2 for a clearer illustration of the EI update procedure between each node during the decoding process. The EI update during the decoding iteration is given in the following, which corresponds to the decoding process given in Section 3.3.

For the *l*th iteration of the first stage decoding, firstly EI is exchanged on the Raptor decoding graph of user 1, i.e., on the upper part of Figure 3:

*Step 1:* The LLR messages are transferred from user 1’s input nodes to the LDPC check nodes. The EI of the messages is:

(16) $$x_{1,\;ext}^{\left(l-1\right)}=\sum_{d=1}^{d_{v}}\alpha_{1,d}J\left(dJ^{-1}\left(x_{1,u}^{\left(l-1\right)}\right)\right),$$

where $x_{1,u}^{\left(l-1\right)}$ is the EI of the messages from the output nodes to the input nodes in the (l−1)th iteration.

*Step 2:* EI from the LDPC check nodes to the input nodes of user 1 is:

(17) $$T\left(x_{1,ext}^{\left(l-1\right)}\right)=\sum_{d=1}^{d_{v}^{\prime }}\xi_{d}J\left(dJ^{-1}\left(1-\sum_{j=1}^{d_{c}^{\prime }}{\tilde{\rho }}_{j}J\left(\left(j-1\right)J^{-1}\left(1-x_{1,ext}^{\left(l-1\right)}\right)\right)\right)\right).$$

*Step 3:* EI from user 1’s input nodes to the output nodes is:

(18) $$x_{1,v}^{l}=\sum_{d=1}^{d_{v}}{\tilde{\alpha }}_{1,d}J\left(\left(d-1\right)J^{-1}\left(x_{u\left(1\right)}^{\left(l-1\right)}\right)+J^{-1}\left(T\left(x_{ext\left(1\right)}^{\left(l-1\right)}\right)\right)\right).$$

*Step 4:* EI from the output nodes of user 1 to the input nodes:

(19) $$x_{1,u}^{\left(l\right)}=1-\sum_{d=1}^{d_{c}}\omega_{1,d}J\left(\left(d-1\right)J^{-1}\left(1-x_{1,v}^{\left(l\right)}\right)+J^{-1}\left(1-I_{DET1}\left(I_{out2}^{\left(l-1\right)};H\right)\right)\right),$$

where $I_{DET1}\left(I_{out2}^{\left(l-1\right)};H\right)$ is the EI of the messages from the MU detector. Its value is determined by the channel matrix H and the EI $I_{out2}^{\left(l-1\right)}$ corresponding to the messages from the output nodes of user 2 in the (l−1)th iteration. The derivation of $I_{DET1}$ will be discussed later.

*Step 5:* EI from LT output nodes of user 1 to the MU detector:

(20) $$I_{out1}^{\left(l\right)}=1-\sum_{d=1}^{c}{\;\Omega }_{1,d}J\left(dJ^{-1}\left(1-x_{1,v}^{\left(l-1\right)}\right)\right).$$

The EI update on the Raptor decoding graph of user 2 is similar, which is omitted here.

**Figure 5:**
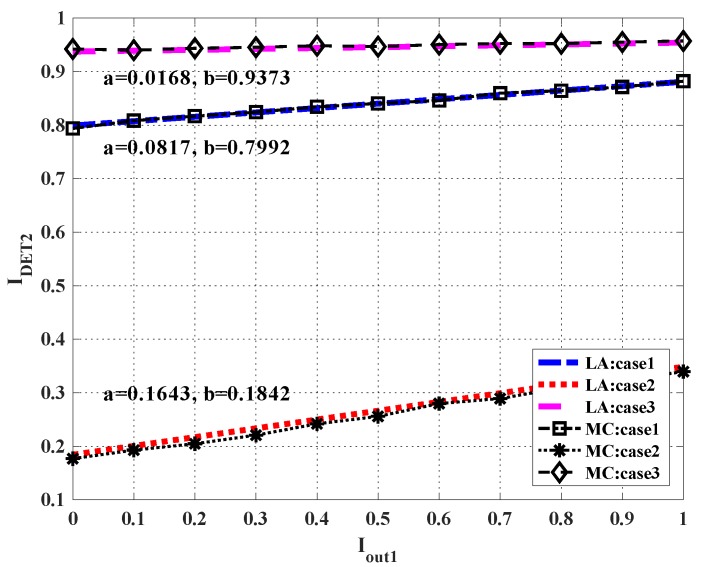
Extrinsic information transfer (EXIT) chart for the multi-user (MU) detector.

In step 4, $I_{DET1}\left(I_{out2};H\right)$ is the output EI from the detector. The input EI is $I_{out2}$, which is the EI from the LT output node of user 2 to the detector. This function can be numerically derived by Monte Carlo simulations [44]. In detail, $N_{mont}$ transmitted symbols $x_{1}$ and $x_{2}$ of user 1 and user 2 are generated, respectively. For user 1, the symbol value is fixed to be 1, while for user 2, the symbol value is randomly 1 or −1. With randomly generated Gaussian noise samples, $N_{mont}$ samples of quantized signals from each RRH can be generated. For each fixed $I_{out2}$, we generate $N_{mont}$ samples of $LLR^{e}\left[c_{2}\right]$ from the symmetric Gaussian distribution with mean τ and −τ, depending on the corresponding $x_{2}$ being either +1 or −1, respectively, where τ is calculated from (14). Then, $N_{mont}$ samples of $LLR^{a}\left[c_{1}\right]$ can be generated. $I_{DET1}$ is evaluated using the histogram for the probability distribution of $LLR^{a}\left[c_{1}\right]$. According to the results of Monte Carlo simulations, we find that under fixed H, $I_{DET1}$ can be well approximated by a linear function:

(21) $$I_{DET1}=aI_{out2}+b,$$

where a and b are coefficients which can be determined by two end points of the line, i.e., $I_{DET1}\left(0;\;H\right)=I\left(x_{1};{\hat{y}}_{1},{\hat{y}}_{2}\right)$ and $I_{DET1}\left(1;\;H\right)=I(x_{2};{\hat{y}}_{1},{\hat{y}}_{2}|x_{1})$. Figure 4 shows an example where $SNR=\frac{P}{\sigma_{0}}$= −2dB, the number of quantization bits at the RRH is eight, three channel matrices **H** are considered: case 1 is $H=\left[\begin{array}{cc} 1.9781 & 0.6696\\ 1.2958 & 0.3872 \end{array}\right]$, case 2 is $H=\left[\begin{array}{cc} 0.5826 & 1.1291\\ 0.8247 & 1.5324 \end{array}\right]$, and case 3 is $H=\left[\begin{array}{cc} 2.8884 & 0.4533\\ 0.3361 & \;1.1801 \end{array}\right]$. From Figure 5, one can find that the linear lines (called LA in the figure) well approximated the Monte Carlo simulations (called MC in the figure).

With step 1 to step 5 shown in the above, we can trace the update of $x_{i,u}^{\left(l\right)}$ in each decoding iteration (recalling that $x_{i,u}^{\left(l\right)}$ is the EI from the output nodes to the input nodes of user *i*). To guarantee the successful decoding at the second decoding stage which is individually performed on the LDPC subgraph, $x_{i,u}^{\left(l\right)}$ should exceed a certain threshold before the maximal decoding iteration times. Therefore, we derive the successful decoding condition: $x_{i,u}^{\left(l^{\prime }\right)}>x_{i,u}^{th}$, where $l^{\prime }$ is the maximal decoding iteration times. Here, the threshold EI $x_{i,u}^{th}$ can be calculated from the following equation (recalling that $m_{th}$ is the minimal LLR required for LDPC successful decoding):

(22) $$J\left(m_{th}\right)=\sum_{d=1}^{d_{v}}\alpha_{i,d}J\left(dJ^{-1}\left(x_{i,u}^{th}\right)\right).$$

### 4.2. Degree Profile Optimization

With the above EXIT analysis, we optimize the degree profile of the Raptor code adopted at each user. The task is to find a uniformly good output degree profile for each user under all possible channel states, with the knowledge of the probability distribution of each channel state.

Recall that the channel matrix H remains invariant within each round of transmission and varies independently round by round. For each round, both users continuously transmit the Raptor codeword until the BBU pool decodes both messages successfully. Hence, the length of the Raptor codeword for each user is related to the channel matrix H in each round, which can be expressed as:

(23) $$L_{i}\left(H\right)=\frac{K}{R_{p}}{\bar{\alpha }}_{i}\left(H\right)\sum_{d=1}^{d_{c}}\frac{\omega_{i,d}}{d},$$

where ${\bar{\alpha }}_{\left(i\right)}\left(H\right)$ is the average degree of the LT input nodes for user *i* under channel matrix H. Note that during each round of transmission, the two users start and stop transmitting simultaneously, therefore we have $L_{1}\left(H\right)=L_{2}\left(H\right)≜L\left(H\right)$.

In general, the channel matrix space is continuous. In practice, we discretize the space into Q states: $H_{q},q=1,\;\ldots ,\;Q$. The probability of each state is denoted as $\mathrm{Pr}\left(H_{q}\right)$. The average Raptor code length over all Q channel states can be expressed as:

(24) $$E\left\{L_{i}\left(H_{q}\right)\right\}=\sum_{q=1}^{Q}\mathrm{Pr}\left(H_{q}\right)\frac{K}{R_{p}}{\bar{\alpha }}_{i}\left(H_{q}\right)\sum_{d=1}^{d_{c}}\frac{\omega_{i,d}}{d}.$$

Apparently, $E\left\{L\left(H_{q}\right)\right\}=E\left\{L_{1}\left(H_{q}\right)\right\}=E\left\{L_{2}\left(H_{q}\right)\right\}$.We can approximate ${\bar{\alpha }}_{i}\left(H_{q}\right)$ as ${\bar{\alpha }}_{i}\left(H_{q}\right)={\bar{\alpha }}_{i,0}C_{i}{}^{-1}\left(H_{q},P\right)$ [45], where ${\bar{\alpha }}_{i,0}$ is a constant and is independent of the channel matrix. $C_{i}\left(H_{q},P\right)$ is the theoretically maximal achievable rate of user i with transmit power P under channel matrix $H_{q}$. $C_{i}\left(H_{q},P\right)$ can be calculated as follows.

**Figure 6:**
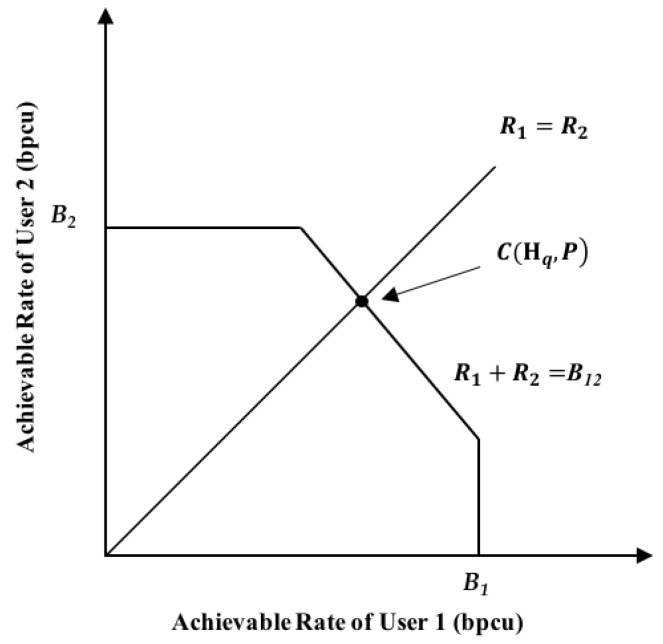
Capacity region of the two-user MIMO system.

According to (1), the two-user C-RAN can be regarded as a two-user multiple-input-multiple output (MIMO) system with two antennas at the receiver. Note that to be precise, the latter is the ideal case of the former wherein the quantization error at RRHs is ignored. The capacity region of the two-user MIMO system is depicted in Figure 6, and can be expressed as:

(25) $$R_{1}\leq I\left(x_{1};y|x_{2}\right)=H\left(y|x_{2}\right)-H\left(y|x_{1},x_{2}\right)≜B_{1},$$

(26) $$R_{2}\leq I\left(x_{2};y|x_{1}\right)=H\left(y|x_{1}\right)-H\left(y|x_{1},x_{2}\right)≜B_{2},$$

(27) $$R_{1}+R_{2}\leq I\left(x_{1},x_{2};y\right)=H\left(y\right)-H\left(y|x_{1},x_{2}\right)≜B_{12},$$

where $R_{1}$ and $R_{2}$ denote the achievable rates of user 1 and user 2, respectively. The derivation of each entropy term is not difficult, thus it is omitted here. Note that in our setting, both users have the same message length and transmit simultaneously (i.e., the times of channel usage for both users are the same). Therefore, the achievable rates of the two users are always the same. The maximal achievable rate for both users should be the intersection point of the line $R_{1}=R_{2}$ and the capacity region boundary, which is given by

(28) $$C_{1}\left(H_{q},P\right)=C_{2}\left(H_{q},P\right)=C\left(H_{q},P\right)≜\max\left(\min\left(B_{1},B_{2},\frac{1}{2}B_{12}\right)\right),$$

where $B_{1}$, $B_{2}B_{2}$ and $B_{12}$ are the right-hand sides of (25), (26) and (27), respectively.

Note that the average network throughput can be expressed as $T=\frac{K}{E\left\{L\left(H_{q}\right)\right\}}$. Thus, it is inverse to the average Raptor code length of each user. Our optimization object is to find the optimal degree profile that minimizes the average code length while guaranteeing successful decoding at the BBU pool under all possible channel states. However, this problem is difficult to solve and the linear programming method in [39] cannot be applied here. We transform the original optimization problem. Instead, we minimize the required SNR for successfully decoding with a fixed average code length. Note that a similar technique has been used in the AWGN case for a multiple access relay channel [44]. In our problem, the fixed average code length is derived as a lower bound of the code length for each user to successfully deliver a message of length K, as given below:

(29) $$L=\frac{K}{R_{p}}\sum_{q=1}^{Q}\mathrm{Pr}\left(H_{q}\right)C^{-1}\left(H_{q},P\right).$$

The optimization problem is given as follows:

(30) $$\underset{P_{th},{\bar{\alpha }}_{i,0},\left\{\omega_{i,d}\right\}}{\min}P_{th}/\sigma_{0}^{2}\;\;\{\begin{array}{l} C1:\sum_{d=1}^{d_{c}}\omega_{i,d}=1,\;i=1,2,\\ C2:\omega_{i,1}>\varepsilon ,\;i=1,2,\\ \begin{array}{l} C3:\;x_{i,u}^{\left(l^{\prime }\right)}>x_{i,u}^{th},\;for\;all\;channel\;matrices\;H_{q},\;i=1,2\\ C4:\;\frac{K}{R_{p}}\sum_{q=1}^{Q}\mathrm{Pr}\left(H_{q}\right){\bar{\alpha }}_{i,0}C^{-1}\left(H_{q},P_{th}\right)\sum_{d=1}^{d_{c}}\frac{\omega_{i,d}}{d}=L,\;i=1,2 \end{array} \end{array}.$$

In the above problem, C2 is the starting condition for BP decoding, where ε is a small number. C3 guarantees successful decoding with the average input node degree ${\bar{\alpha }}_{i}\left(H_{q}\right)$ and degree profiles $\left\{\omega_{i,d}\right\}$ under all channel states, where *l*’ is the maximal decoding iteration times. C4 is from (24) and (29), i.e., the average code length is fixed to *L*. We solve the above optimization using the differential evolution (DE) method [46]. The common DE includes mutation, crossover and selection operations. The procedure is as follows:

(1) Initialization: We first initialize the vector population $\left\{\omega_{a}\left(0\right)|a=1,2,\ldots ,NP\right\}$ as the 0th generation, where each vector individual $\omega_{a}\left(0\right)≜\left[\omega_{a,1}\left(0\right),\omega_{a,2}\left(0\right),\ldots ,\omega_{a,d_{c}}\left(0\right)\right]$ represents one degree profile, and NP is the number of individuals. $\omega_{a,b}\left(0\right)$ represents the coefficient for degree *b* in the profile, and dc is the maximal degree. $\omega_{a}\left(0\right)$ should satisfy the conditions of C1 and C2 in the problem (30).
(2) Mutation: For the Gth generation, the mutation vector of the next generation can be generated by
  (31) $$v_{a}\left(G+1\right)=\omega_{r_{1}}\left(G\right)+F\cdot \left(\omega_{r_{2}}\left(G\right)-\omega_{r_{3}}\left(G\right)\right),\;a\neq r_{1}\neq r_{2}\neq r_{3},a=1,\ldots ,NP,$$
  where F∈(0,1] is a scaling factor (we choose F=0.5 in this paper), and $r_{1}$, $r_{2}$ and $r_{3}$ are random integers between 1 and *NP*.
(3) Crossover: Cross $\omega_{a}\left(G\right)$ and $v_{a}\left(G+1\right)$ to get (where a=1,2,…,NP, $b=1,\ldots ,d_{c}$*)*:
  (32) $$u_{a,b}\left(G+1\right)=\{\begin{array}{l} v_{a,b}\left(G+1\right),\;if\;rand\left(0,1\right)\leq CR\;or\;b=b_{rand}\\ \omega_{a,b}\left(G\right),\;otherwise \end{array},$$
  where CR is the crossing probability (we choose CR=0.5 in this paper) and $b_{rand}$ $\in \left[1,\ldots ,d_{c}\right]$ is a random integer.
(4) Selection: We select individuals for the next generation by (where a=1,2,…,NP):
  (33) $$\omega_{a}\left(G+1\right)=\{\begin{array}{l} u_{a}\left(G+1\right),\;if\;F\left(u_{a}\left(G+1\right)\right)\leq F\left(\omega_{a}\left(G\right)\right)\\ \omega_{a}\left(G\right),\;otherwise \end{array},$$
  where the value of function F(·) stands for the threshold SNR $P_{th}/\sigma_{0}^{2}$ for successful decoding. If the minimal $P_{th}/\sigma_{0}^{2}$ does not change within a certain number of iterations, the algorithm ends. The degree profile corresponding to the smallest $P_{th}/\sigma_{0}^{2}$ is selected as the optimal edge degree profile $\left\{\omega_{i,d}\right\}$. Finally, according to (15), the optimal output node degree distributions $\Omega_{i,\mathrm{opt}}\left(x\right)$ are derived.

## 5. Simulation Results

In this section, we simulate the bit error rate (BER) and the achieved throughput of the proposed transmission scheme. In the simulation, we consider the scenario where two users in C-RAN are located within the coverage of two RRHs. The SNR is defined as the ratio of the user-transmitted power P to the Gaussian noise variance $\sigma_{0}^{2}$ at each RRH. We assume that the channel matrix **H** has three states with equal probability: $H_{1}=\left[\begin{array}{cc} 1.9781 & 0.6696\\ 1.2958 & 0.3872 \end{array}\right]$, $H_{2}=\left[\begin{array}{cc} 0.5826 & 1.1291\\ 0.8247 & 1.5324 \end{array}\right]$, $H_{3}=\left[\begin{array}{cc} 2.8884 & 0.4533\\ 0.3361 & \;1.1801 \end{array}\right]$. Two cases for the quantizer at the RRHs are considered, wherein the number of quantization bits are three and eight, respectively. Apparently, the latter case generates more fronthaul traffic than the former. We assume that the length of message for each user is *K =* 9500 bits, that both users adopt the same LDPC code with $R_{p}=0.95$ and that the decoding threshold for the LDPC code is derived as $m_{th}=13.6359$.

(1) BER performance

Firstly, we simulate the error performance of the proposed scheme versus the decoding overhead under each of the channel states $H_{1}$, $H_{2}$ and $H_{3}$, respectively. We fix P=0.84 and $\sigma_{0}^{2}=1.2^{2}$. The decoding overhead is defined as $overhead=\frac{C\left(H_{q},P\right)\cdot N}{K}-1$, where $C\left(H_{q},P\right)$ is the theoretical upper bound of the achievable rate of the user (recalling (30)), N is the actual transmitted code length. A larger overhead means a longer code length N and a lower achieved rate. The overhead of value 1 can serve as the theoretical limit. We optimize the degree profile for each user using the scheme proposed in Section 4.2. The resulted profiles are as follows:

When an eight-bit quantizer is adopted at each RRH, the optimized degree profiles for the two users are denoted as $\Omega_{1,opt,8-bit}\left(x\right)$ and $\Omega_{2,opt,8-bit}\left(x\right)$:

$$\Omega_{1,opt,8-bit}\left(x\right)=0.0120x^{1}+0.4054x^{2}+0.2678x^{3}+0.2217x^{6}+0.0931x^{20},\;\Omega_{2,opt,8-bit}\left(x\right)=0.0121x^{1}+0.4011x^{2}+0.2808x^{3}+0.1564x^{6}+0.0401x^{7}+0.1004x^{18}+0.0090x^{19}.$$

When a three-bit quantizer is adopted, the optimized degree profiles for the two users are:

$$\Omega_{1,opt,3-bit}\left(x\right)=0.0120x+0.4028x^{2}+0.2693x^{3}+0.2020x^{6}+0.0231x^{7}+0.0907x^{20},\;\Omega_{2,opt,3-bit}\left(x\right)=0.0125x+0.4151x^{2}+0.2053x^{3}+0.1042x^{5}+0.2020x^{6}+0.1025x^{20}.$$

**Figure 7:**
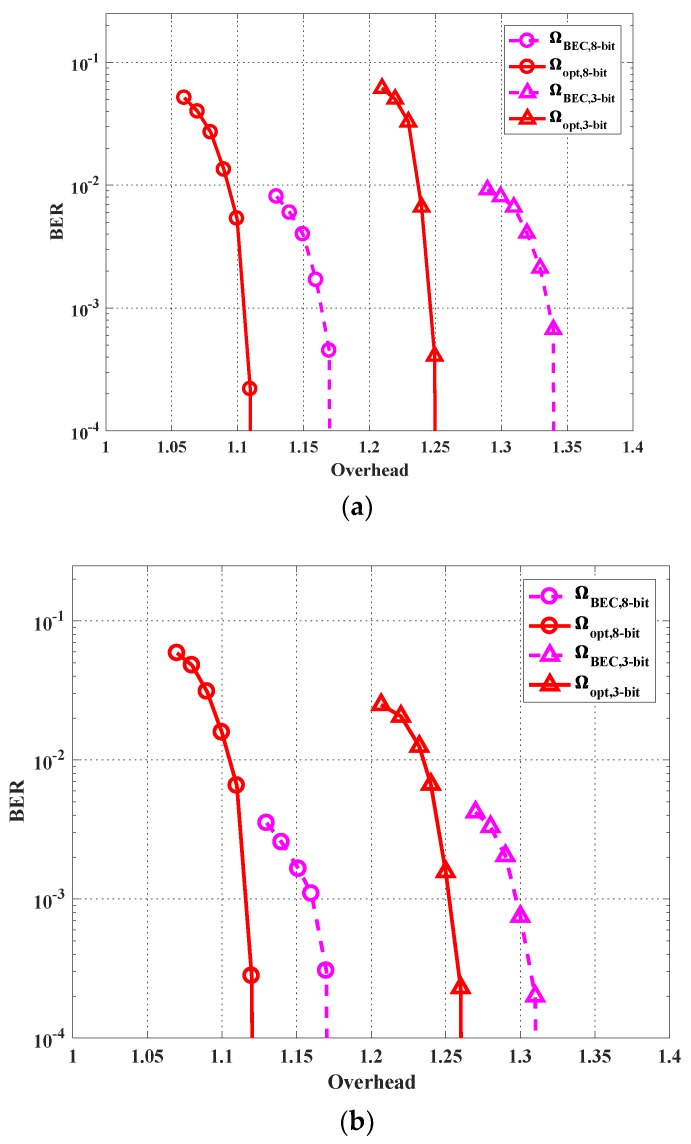

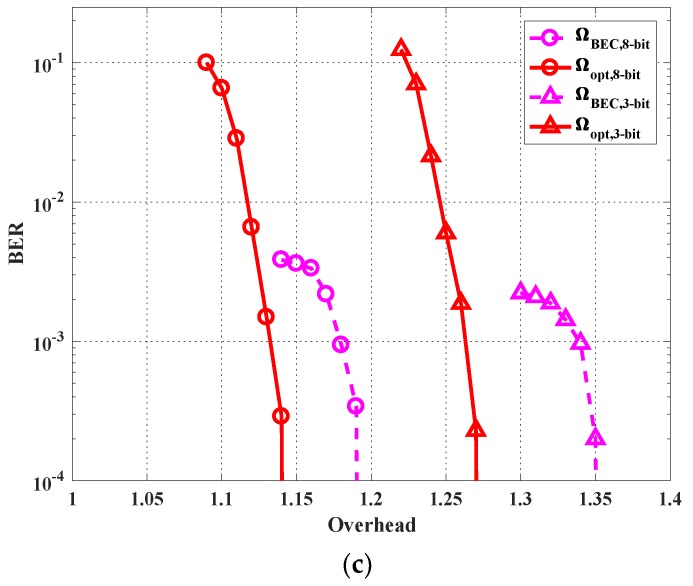
Bit error rate (BER) performance with the optimized profiles and benchmark profile for the cases of the three-bit quantizer and the eight-bit quantizer under different channel matrices: (**a**) $H_{1}$, (**b**) $H_{2}$, and (**c**) $H_{3}$.

As for comparisons, we also simulate the performance of the degree profile $\Omega_{BEC}\left(x\right)$ (which is optimal under binary erasure channel (BEC) [43]):

$${\;\Omega }_{BEC}\left(x\right)=0.0008x+0.494x^{2}+0.166x^{3}+0.073x^{4}+0.083x^{5}+0.056x^{8}\;+0.037x^{9}+0.056x^{19}+0.025x^{65}+0.003x^{66},$$

for both three-bit and eight-bit cases. In Figure 7, the three subfigures (a), (b) and (c) show the BER performance of the optimized profiles and BEC profile under channel matrices $H_{1}$, $H_{2}$ and $H_{3}$, respectively. Interestingly, although the degree profiles are optimized to improve the average performance under the three channel states, they still perform well under each channel state. For both three-bit and eight-bit cases, the optimized profiles achieve a BER under $10^{-4}$ at a smaller overhead compared with the BEC profile. It is also observed that the eight-bit quantizer case has a lower BER compared with the three-bit quantizer case, which verifies the fact that a quantizer with less quantization bits introduces larger quantization noise. Finally, it can be seen that under all the three channel states, the optimized profile under the eight-bit case achieves $10^{-4}$ BER at an overhead of less than 1.14, which is close to the theoretical limit (which ignores the quantization noise), i.e., overhead=1.

(2) Throughout performance

**Figure 8:**
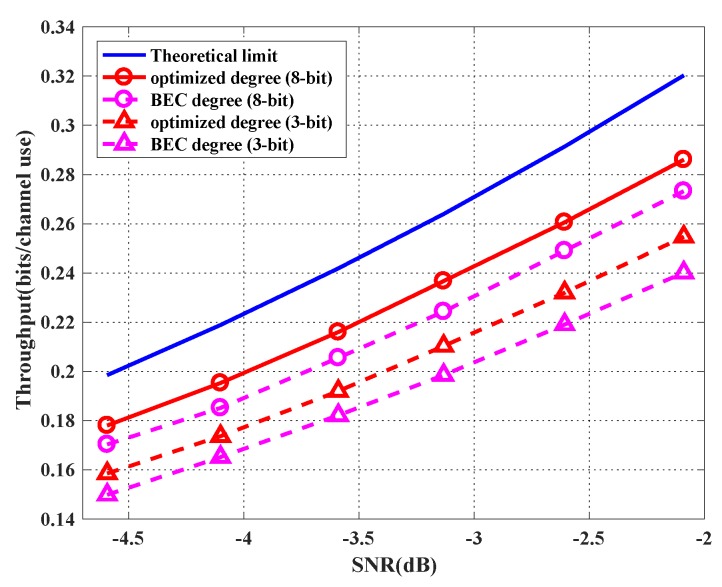
Achieved throughput with the optimized profiles and benchmark profile for the cases of the three-bit quantizer and the eight-bit quantizer.

We simulate the average sum throughput under different SNRs. We fix $\sigma_{0}^{2}=1.2^{2}$ but change the transmission power P for different SNRs. For each SNR, we optimize the degree profiles of the two users for both three-bit and eight-bit quantizer cases. Figure 8 shows the simulation results. For each point in the figure, we simulate 100 rounds of transmission, and the channel matrix is uniformly and randomly chosen from $H_{1}$, $H_{2}$ and $H_{3}$ in each round. As can be seen in Figure 7, the optimized degree profile achieves a higher throughput compared with the BEC profile. The performance of the eight-bit quantization case with the optimized degree profiles is within only 11% away from the theoretical limit. Here, the theoretical limit is the upper bound of the average theoretically achievable sum rate, which is calculated by

(34) $$C_{lim}=\frac{1}{\sum_{q=1}^{3}C^{-1}\left(H_{q},P\right)\mathrm{Pr}\left(H_{q}\right)}.$$

## 6. Conclusions

We considered the rateless coded transmission scheme for two-user uplink in C-RAN with two RRHs under a block fading channel. In the proposed scheme, both users keep transmitting until the BBU pool feeds back ACKs, where instant CSI is not required by the users. We also proposed a scheme to find the universally good degree profiles for both users under all possible channel states, with only statistics information of the channel matrix. In detail, the degree profiles are optimized to minimize the threshold SNR under the conditions that the average code length is fixed and the message of each user can be correctly recovered at the BBU pool in all possible channel states. It can be seen from the simulation that the optimized degree profiles have better performance for both BER and system throughput, compared with the BEC profile. Moreover, the achieved throughput has only about 11% loss from the theoretical limit.

Note that although only a two-user case is considered in this paper, the extension to the multi-user case is not difficult. One can simply extend the iterative receiver structure in Figure 2, where the output LLR of the MU detector can be calculated accordingly. Using EXIT analysis, the degree profile optimization problem can also be formulated, and DE can be applied.

## Figures and Tables

**Table 1:** Annotations to symbols.

Symbol	Annotation
ξ(x)	LDPC variable node degree distribution
$\xi_{d}$	Probability of an LDPC variable node with degree d
$\tilde{\rho }\left(x\right)$	Edge distribution of the LDPC check nodes
$\;{\tilde{\rho }}_{d}$	Probability of an edge connected to an LDPC check node with degree d
$\alpha_{i}\left(x\right)$	Input node degree distribution for user *i*
$\alpha_{i,d}$	Probability of input nodes with degree d for user *i*
${\tilde{\alpha }}_{i}\left(x\right)$	Edge distribution of the input nodes for user i
${\tilde{\alpha }}_{i,d}$	Probability of an edge connected to an input node with degree d for user *i*
$\omega_{i}\left(x\right)$	Output node edge distribution for user i
$\omega_{i,d}$	Probability of an edge connected to an output node with degree d of user i
$d_{v}$	Maximum degree of input nodes
$d_{v}^{\prime }$	Maximum degree of LDPC variable nodes
${\bar{\alpha }}_{i}$	Average degree of the input nodes for user i
